# Direct analysis of thymic function in children with Down's syndrome

**DOI:** 10.1186/1742-4933-2-4

**Published:** 2005-02-16

**Authors:** Nicole Prada, Milena Nasi, Leonarda Troiano, Erika Roat, Marcello Pinti, Elisa Nemes, Enrico Lugli, Roberta Ferraresi, Luigi Ciacci, Davide Bertoni, Ornella Biagioni, Milena Gibertoni, Cristina Cornia, Liviana Meschiari, Elisabetta Gramazio, Mauro Mariotti, Ugo Consolo, Fiorella Balli, Andrea Cossarizza

**Affiliations:** 1 Dipartimento di Biopatologia e Metodologie Biomediche, Università di Palermo, via Tukory 211, 90134 Palermo, Italy; 2 Cattedra di Immunologia, Dipartimento di Scienze Biomediche, Università di Modena e Reggio Emilia, via Campi 287, 41100 Modena, Italy; 3 Clinica Odontoiatrica, Università di Modena e Reggio Emilia, via del Pozzo 71, 41100 Modena, Italy; 4 Servizio di Neuropsichiatria Infantile, AUSL Modena, via Cardarelli 45, 41100 Modena, Italy; 5 Clinica Pediatrica, Università di Modena e Reggio Emilia, via del Pozzo 71, 41100 Modena, Italy

**Keywords:** Down's syndrome, thymus, TREC, T lymphocytes

## Abstract

**Background:**

Down's syndrome (DS) is characterized by several immunological defects, especially regarding T cell compartment. DS is considered the best example of accelerated ageing in humans. Direct observations of the thymus have shown that in DS this organ undergoes severe histological and morphological changes. However, no data on its capacity to generate T cells are present in the literature. Here, using a new technology based upon real time PCR, we have investigated the capacity of the thymus to produce and release newly generated T lymphocytes (the so called "recent thymic emigrants", RTE) in children with DS.

**Methods:**

We studied 8 children affected by DS, aged 2–7 years, compared with 8 age- and sex-matched healthy controls. Flow cytometry was used to determine different lymphocytes subsets. Real time PCR with the Taqman system was used to quantify the amount of RTE, *i.e. *peripheral blood lymphocytes that express the T cell receptor rearrangement excision circles (TREC).

**Results:**

In comparison with control children, those with DS had a significant lower number of TREC+ peripheral blood cells. Moreover, in DS children but not in controls, a strong negative correlation between age and the levels of TREC+ cells was found.

**Conclusions:**

The direct measure of thymic output indicates that the impairment of the organ results in a reduced production of newly generated T cells. This observation could suggest that cytokines able to modulate thymic function, such as interleukins, could be useful to improve the functionality of the organ and to treat the immunodeficiency present in DS subjects.

## Introduction

Down's Syndrome (DS) is the most common chromosomal abnormality in humans, that occurs in 1 out of every 800–1,000 births. It is an autosomal disorder resulting by triplication of chromosome 21. Many characteristics are commonly seen in DS, including some degree of intellectual impairment, that varies widely from individual to individual, heart defects, hypotonia, hyperuricemia, and the development of Alzheimer disease-type neuropathology beginning at about 40 years of age [1].

DS subjects also present alterations of the immune system which are similar to those of aged people, including increased susceptibility to bacterial and viral infection and to the onset of different types of haematological malignancies, along with a high frequency of autoantibodies. Alterations of B lymphocytes, T cell subsets, and natural killer cells, defective phagocytosis and chemotaxis of polymorphonuclear leukocytes and interleukin-2 production by activated T cells were also reported [2-7]. Quantitative studies of peripheral blood T lymphocytes reveal a reduction, often quite small, in the percentage and/or absolute number of T lymphocytes, although normal proportions or numbers of T and B lymphocytes in DS children have also been reported [8].

Several studies have focused their attention on the role of the thymus, and have described a variety of structural and anatomic alterations present in DS [9]. Few data, if any, exist on the direct measure of thymic functionality in terms of production of newly generated T cells. Accordingly, the aim of this study was to measure the capability of the thymus of DS children to produce new T lymphocytes, and to analyze how such capability changes with age. For this reason, we have quantified the so called "recent thymic emigrants" (RTE), that are the main contributors to the naïve T-cell pool, and are characterized by the presence in the nucleus of a circular, episomal DNA molecule called TREC (T cell receptor Rearrangement Excision Circles), generated during the intrathymic rearrangement of the α-chain locus of the T cell receptor (TCR).

Genes for the δ-chain of the TCR are distributed within the genomic region that codifies for the α-chain, and are removed in two steps during the recombination of Vα with Jα. Thus, a thymocyte that starts to rearrange the α-chain produces the first TREC, called signal-joint (sj)-TREC, then proliferates three or four times, and finally completes the rearrangement of Vα with Jα, so producing the second TREC, called coding-joint (cj)-TREC. The removal of genes for the δ-chain from the α region does not imply their elimination, as such DNA remains into the nucleus as a circle that is not able to replicate. As a consequence, when a cell undergoes a division, TREC are passed only to one of the two daughter cells. During the following cell cycles, TREC are then diluted into the population that origins from the first cell. Several data indicated that the percentage of TREC+ cells is a marker of thymic activity, that TREC+ cells are almost present within the subset of virgin T lymphocytes, and that their number consistently declines with age [10].

## Materials and Methods

### Subjects

We could analyze blood samples from 8 children with DS (with a mean age of 4.62 years, range: 2–7 years), 7 males and 1 females. As control group, we studied 8 children with a mean age of 4.75 years (range, 2–8 years), 7 males and 1 female. All subjects were in good conditions, and had no acute or chronic disease affecting the immune system. Written informed consent was obtained by their parents, according to the Italian laws.

### Isolation of PBMC from blood

Peripheral blood mononuclear cells (PBMC) were separated from a minimum of 8 mL freshly collected blood according to standard procedures.

### Analysis of the phenotype of peripheral blood lymphocytes

Blood samples were stained with different monoclonal antibodies for the cytometric analysis, as described [11]. The quantification of the main subpopulations present among lymphocytes (electronically selected on the basis of an electronic gate put in the region of lymphocytes) was performed by flow cytometry using a CyFlow ML instrument (Partec, Münster, Germany), according to standard procedures [11].

### DNA extraction

DNA was extracted from 5 × 10^6 ^PBMC using the QIAamp DNA Mini Kit (QIAGEN) according to the manufacturer's protocols and stored in sterile water at -20°C until use.

### Quantification of percentage of sjTREC positive PBMC by a Real Time PCR approach

The percentage of PBMC containing sjTREC was measured by an original method that has been recently developed (patent pending) using a Real Time PCR approach. This assay was performed by two parallel polymerase chain reactions (PCR), that quantify sjTREC or nuclear DNA (nDNA) in a given sample, carried out in two different reaction tubes, but in the same plate, in order to have similar reaction conditions.

In the first reaction, we quantified sjTREC using a mix that consisted of: Supermix Biorad 1X, primers for sjTREC 500 pmol and we added 3 μl of the sample in each tube. The sjTREC primers we used were: sjTREC Dir (5'-CAC ATC CCT TTC AAC CAT GCT-3') and sjTREC Rev (5'-GCC AGC TGC AGG GTT TAG G-3'). TaqMan probe for sjTREC (5'-FAM-ACA CCT CTG GTT TTT GTA AAG GTG CCC ACT – BLACK HOLE-3') was included in the reaction mixture at the concentration of 200 nM, as a real time detector for the amplified product. One cycle of denaturation (95°C for 6 min) was performed, followed by 50 cycles of amplification (95°C for 30 sec, 58°C for 1 min 30 sec). The same approach was used to quantify nDNA, which was required to obtain the number of cells present in the sample. In this case, primers GenDir (5'-GGC TCT GTG AGG GAT ATA AAG ACA-3') and GenRev (5'-CCA ACC ACC CGA GCA ACT AAT CT-3'), designed on FasL gene sequence, present in two copies in the human genome (one in each chromosome 1), were used at the concentration of 600 and 400 nM respectively, and the TaqMan probe GenProbe (5' TexasRed – CTG TTC CGT TTC CTG CCG GTG C – BlackHole Quencher2 3') was included in the reaction mixture at the concentration of 300 nM. One cycle of denaturation (95°C for 3 min 30 sec) was performed, followed by 45 cycles of amplification (95°C for 30 sec, 60°C for 35 sec).

All of the aforementioned primers and probes have been designed using the program "primer3", available on the internet address: . The length of amplified fragments is 104 pb for nDNA and 101 pb for sjTREC.

PCR was performed by using an iCycler Thermal cycler (BioRad, Hercules, CA, USA), that monitors changes in the fluorescence spectrum of each reaction tube during the annealing phase. The fluorescence signal was processed using the "Real-time detection system iCycler iQ" software, that calculates the threshold and the threshold cycles of each sample. All reactions were carried out in triplicate.

To optimize the precision of the assay, whose results derive from the comparison between the threshold cycles of two different real time PCR reactions, we have developed a new approach. Indeed, the regions used as template for the two amplifications (*i.e.*, those of sjTREC and nDNA) were purified and cloned tail to tail in a vector (pGEM-11Z, from Promega), to have a ratio of 1:1 of the molecules used as reference.

The nDNA region used has been excised as a SacI-ApaI fragment and cloned in the pGEMT easy vector (Promega), obtaining the pGEM11Z-nDNA vector. The sjTREC region used has been excised as a PstI-SacI fragment and cloned in the same vector, obtaining the pSJ vector we used. The plasmid has been purified, quantified with the spetrofotometer and linearized using SacI. Then a series of reactions has been performed for nDNA and sjTREC with serial dilution of the standard. All the times the threshold cycles of nDNA and sjTREC were the same, proving the ratio 1:1 of the two fragments.

Then, serial known dilutions of this vector, amplified in triplicate, were included in each PCR run to generate a standard curve from which the relative copy number of either sjTREC or nDNA present in the unknown samples was determined. The measured values for sjTREC and nDNA were always within the range of the standard curve, whose correlation coefficient was always >0.990. The values of sjTREC and nDNA present in each sample were calculated using the mean of the threshold cycles of the three replicates.

Then, the percentage of PBMC containing sjTREC in each sample was simply obtained from the ratio between the relative values of sjTREC (obtained dividing for 3 the result given by the iCycler, as we used 3 μl of DNA of the sample and 1 μl of standard to quantify sjTREC) and nDNA obtained (obtained *versus *the same vector), multiplied by 2 (as two copies of the nuclear gene are present in a cell).

## Results

### Peripheral blood cell phenotype in DS

**Figure 1:**
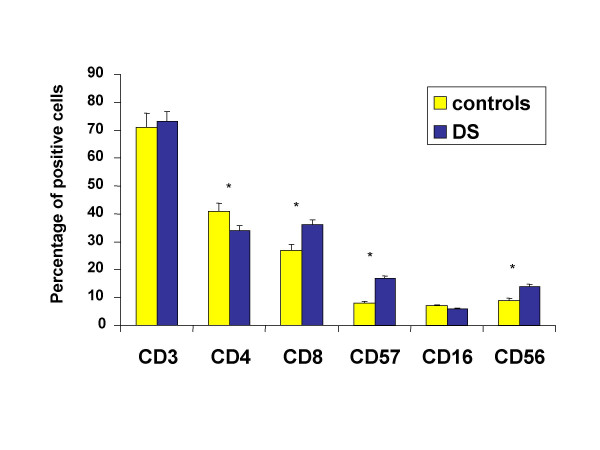
Phenotypic analysis of peripheral blood lymphocytes in patients with Down's syndrome (DS) and healthy subjects. Data are referred to 8 individuals per group. Asterisk indicates a statistically significant difference (p < 0.05).

Down's syndrome is characterized by severe immunological alterations, which mainly affect the T cell compartment and are often regarded as signs of accelerated ageing. Numerous reports suggested that thymic retention of T cells or maturation defects might be the cause of the observed alterations in the T cell compartment. Accordingly, we first checked the presence of possible changes in the phenotype of peripheral blood lymphocytes from DS children. As shown in Figure 1, we found that, in comparison with karyotypically normal healthy controls, DS children had less T helper cells and more cytotoxic T lymphocytes or cells expressing markers related to NK activity. This indicates that the population we analyzed can well represent the immunological situation present in DS children.

### Quantitative analysis of cells expressing TREC

**Figure 2:**
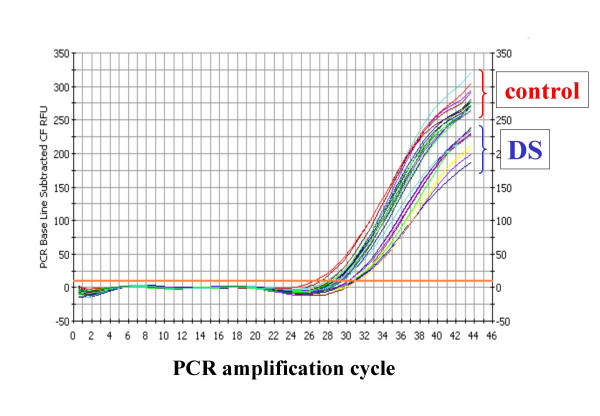
Representative example of real time PCR for the quantification of TREC in a group of 3 children with Down's syndrome (DS) and in 4 healthy controls (each measure is performed in triplicate). Note how the threshold cycle is different in the two groups.

We then studied directly the capacity of the thymus to produce new T lymphocytes, by the analysis of TREC+ cells. Figure 2 shows a typical example of real time PCR assay for quantification of the amount of TREC per cell. It is to note that the threshold cycle, *i.e. *the cycle of PCR in which the fluorescent signal deriving from the amplification of the DNA becomes evident, is quite different in DS children and controls. Indeed, such cycle is much lower in control children, indicating the presence of a higher number of TREC.

**Figure 3:**
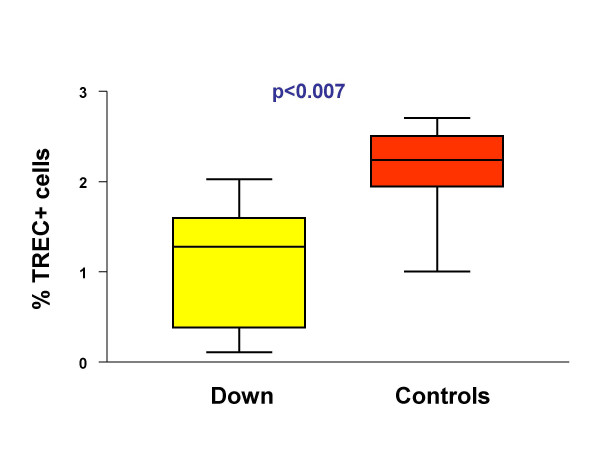
DS children have less TREC+ lymphocytes than healthy controls, as shown in this box-and-whiskers graphics. The boxes extend from the 25^th ^percentile (x_[25]_) to the 75^th ^percentile (x_[75]_) [*i.e.*, the interquartile range (IQ)]; lines inside boxes represent median values. Lines emerging from boxes (*i.e.*, the whiskers) extend to the upper and lower adjacent values. The upper adjacent value is defined as the largest data point ≤x_[75]_+1.5xIQ, and the lower adjacent value is defined as the smallest data point ≥x_[25]_-1.5xIQ. Note that no outliers are present in the two groups.

The percentage of TREC+ lymphocytes in children affected by Down's syndrome and in controls is reported in Figure 3. A statistically significant difference was present between the two groups (p = 0.007), indicating that DS is characterized by a lower thymic output.

### Correlation between age and TREC

**Figure 4:**
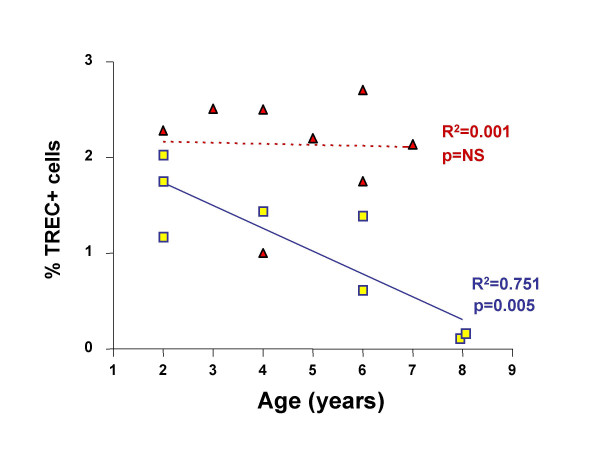
Correlation between age and TREC levels in DS (squares) and control (triangles) children. In the year range 2–8, the correlation was significant in DS but not in control children.

Finally, we have investigated the correlation between age and TREC levels in the two groups. As reported in Figure 4, it is noteworthy that while control children did not display any age-related change (in the age range 2–8 years), those with DS had a significant age-related decrease in the number of TREC+ cells. This indicates that in DS, in contrast with control children, major changes in thymic output occur in the very first years of life.

## Discussion

Immunological ageing is part of a continuum of developmental processes, encompassing complex reorganizational events, compensatory mechanisms and qualitative alterations in the functionality of several systems and organs. Among those organs that undergo major changes with ageing, thymus plays a special role [12]. The thymus is a central lymphoid organ that is the primary site of T-cell maturation and development. Shortly after birth, the thymus undergoes a life long process of involution whereby the organ is replaced by adipose tissue. The result is a reduction in the number of constituent thymocytes with age, a consequent shrinking of the thymus and a decline in the output of newly generated T lymphocytes [13].

Aged peripheral T-cell pool is characterized by the accumulation of T-cell capable of limited replication [14]. Since an efficient immune response is based upon the expansion of antigen-specific clones, the consequence of a qualitative and quantitative impairment of the system is an increased susceptibility to infections or cancer. Several groups, including ours, have studied the immune system during human ageing, using different models, including the one that represents the best example of successful ageing, *i.e. *healthy centenarians, and conditions of accelerated ageing, such as DS [2-6,15].

In DS subjects, the proportion of T-helper cells is decreased, resulting in a decreased ratio of helper/cytotoxic cells, and we could observe this phenomenon also in the patients here described. Furthermore, peripheral blood T cells have a decreased number of cells expressing the T-cell receptor-αβ (TCRαβ) complex, elevated numbers expressing TCRγδ and a decreased proportion of CD4+, CD45RA+ naïve T cells, along with a high number of cells with NK phenotype, suggesting that the DS thymus is inefficient in the release of functionally mature T cells [16]. An age-dependence in the proliferative response to phytohemagglutinin (PHA) of DS lymphocytes, but not of lymphocytes from healthy individuals, was observed. Allogeneic mixed lymphocyte proliferative responses are decreased, as are PHA-induced interleukin-2 production and cytotoxic T-lymphocyte activity [7].

All of the above mentioned alterations have a common substrate, *i.e. *the dysfunction of thymus. Indeed, defects in the capacity to produce thymic hormones has been described several years ago [17,18]. Furthermore, anatomic studies gave further evidence that changes in the T-lymphocyte system derive from structural abnormalities of the thymus. In comparison to age-matched controls, thymuses from infants with DS from 1 day to 15 months of age have marked lymphoid depletion, with a thin cortex and poor corticomedullary demarcation. The Hassall corpuscles are increased in size and frequently cystic. The presence of lower proportions of cells bearing high levels of the TCR-αβ complex and of CD3, a signal-transducing complex for the TCR, in thymuses of children with DS and the increase in the proportions of cells with these markers with age are indicative of delayed maturation of T cells within the thymus [9]. In addition, DS thymuses contain elevated levels of IFN-γ and TNF-α mRNA expressing cells, and there is mast cell hyperplasia and overexpression of class I MHC, CD18 and ICAM-1 [19]. DS thymocytes also have a greater than normal sensitivity to inhibition of IL-4-induced proliferation by IFN-γ and TNF-α [20]. Taken together, these findings indicate an abnormal thymocyte maturation and cytokine dysregulation in the DS thymus, possibly initiated by gene dose-related increased sensitivity to IFN-γ and to overexpression of CD18 (LFA-1β).

In conclusion, in this paper we show that the aforementioned alterations of the thymus result in the reduced production and output of newly generated lymphocytes, that can be directly measured by the assay we have developed and used. The quantitative analysis of TREC+ cells in the periphery is a relatively new and sensitive marker of thymic functionality, which is able to provide information on the status of the organ in different pathological conditions, and on its capability to generate new T cells [21-25]. A markedly reduced capacity of the thymus to produce RTE is present in the DS children we have studied. Such observation, if confirmed in a higher number of cases, could be useful to develop novel strategies to treat the immunodeficiency typical of this syndrome, based for example on the use of cytokines such as interleukin-2 or interleukin-7, which is capable of maintaining or restoring an efficient thymic output [26].
